# Stroke Lateralization in Large Hemisphere Infarctions: Characteristics, Stroke-Related Complications, and Outcomes

**DOI:** 10.3389/fneur.2021.774247

**Published:** 2021-12-10

**Authors:** Jie Li, Ping Zhang, Yingying Liu, Wanli Chen, Xingyang Yi, Chun Wang

**Affiliations:** Department of Neurology, People's Hospital of Deyang, Deyang, China

**Keywords:** hemispheric difference, complications, malignant brain edema, cardiovascular events, outcome, large hemispheric infarction

## Abstract

**Objectives:** To assess the hemispheric differences in characteristics, stroke-related complications, and outcomes of patients with large hemisphere infarctions (LHI).

**Methods:** We enrolled consecutive patients admitted within 24 h after the diagnosis of LHI (defined as an ischemic stroke involving more than 50% of the territory of the middle cerebral artery in computed tomography and/or magnetic resonance imaging). Univariate and multivariate analysis were performed to explore the association between lateralization and stroke-related complications and clinical outcomes.

**Results:** A total of 314 patients with LHI were enrolled, with 171 (54.5%) having right hemispheric involvement. Right-sided patients with LHI had lower baseline National Institutes of Health Stroke Scale (NIHSS) score (18 vs. 22, *p* < 0.001), higher frequency of atrial fibrillation (69.0 vs. 52.4%, *p* = 0.003), and higher proportion of cardio-embolism (73.1 vs. 56.6%, *p* = 0.013) than the left. Right-sided LHI had higher incidence rates of malignant brain edema (MBE) (48.5 vs. 30.8%, *p* = 0.001) and a composite of cardiovascular events (29.8 vs. 17.5%, *p* = 0.011) during hospitalization. The incidence rate of 1-month mortality (34.5 vs. 23.8%, *p* = 0.036) was higher in right-sided patients with LHI, but there were no hemispheric differences in the incidence rates of 3-month mortality and unfavorable outcome (both *p* > 0.05). Multivariate analyses suggested right hemisphere involvement was independently associated with increased risk of MBE (adjusted OR 2.37, 95% CI 1.26–4.43, *p* = 0.007) and composite of cardiovascular events (adjusted OR 2.04, 95% CI 1.12–3.72, *p* = 0.020). However, it was not independently associated with 1-month death, 3-month mortality, and 3-month unfavorable outcome (all *p* > 0.05).

**Conclusions:** Right-sided patients with LHI had higher frequency of atrial fibrillation and cardio-embolism than the left-sided patients. Right hemisphere involvement was independently associated with increased risk of MBE and composite of cardiovascular events during hospitalization, whereas stroke lateralization was not an independent predictor of mortality and unfavorable outcome in patients with LHI.

## Introduction

Large hemispheric infarction (LHI), which usually results from occlusion of the internal carotid artery or proximal middle cerebral artery (MCA), is a devastating condition with a high mortality rate (1, 2). LHI is commonly associated with varying degrees of brain swelling, with subsequently raised intracranial pressure, midline shift, and brain herniation, giving rise to the term malignant brain edema (MBE) (3). Until recently, no pharmacological strategies have been proven effective by clinical trials (4). Decompressive hemicraniectomy (DHC) conducted within 48 h after symptom onset has been proven effective for patients with LHI with MBE (5). However, only 0.3% of highly selected ischemic stroke patients would be eligible for DHC based on the strict eligibility criteria in the DHC trials (6).

It has also been reported that that poststroke complications are the leading cause of death and unfavorable outcomes in ischemic stroke patients (7, 8). Roth et al. have indicated that neurological impairment level is the most substantial factor predicting the rate of complications (9). Our previous work has demonstrated that stroke-related complication occurred in more than three fourths of the patients with LHI and was related to unfavorable outcome, whereas only MBE and pneumonia are independent predictors of a 3-month unfavorable outcome (10). As we know, the NIHSS score are weighted toward left hemisphere lesions (11). It is reasonable to suspect that stroke-related complications might frequently occur in left hemisphere stroke and result in poor outcomes. However, previous works have found that MBE seemed to be more common in the right hemisphere (12). Meanwhile, there is no consensus on the impact of the stroke hemisphere on outcomes of AIS (12–15).

Nowadays, limited data exist regarding the hemispheric differences in the incidence of stroke-related complications and outcomes of patients with imaging-diagnosed LHI. Therefore, we conducted a retrospective cohort study using the prospective data of Deyang stroke registry to assess the hemispheric differences in characteristics, stroke-related complications, and outcomes of patients with LHI.

## Methods

### Study Design and Subjects

From January 1, 2016 to March 31, 2019, patients who were admitted to the Department of Neurology, People's Hospital of Deyang City with either a first-ever or recurrent acute ischemic stroke (AIS) were prospectively and consecutively registered. We enrolled patients who were admitted within 24 h from symptoms onset and diagnosed with LHI. LHI was defined as an infarction in the territory of the MCA, with computed tomography (CT) and/or magnetic resonance imaging (MRI) evidence of infarction that affected more than 50% of the territory of the MCA (with or without the involvement of other vascular territories) during hospitalization, and also an acute onset of corresponding clinical signs and symptoms (16). All patients had a non-contract CT (NCCT) scan before initial treatment. A routine follow-up NCCT or MRI scan was performed within the first 7 days of hospitalization. Other CT scans were performed in case neurological deterioration occurred to determine brain edema or hemorrhagic transformation. We excluded patients with bilateral hemisphere involvement. Cases with incomplete hospital records or missing imaging that would prevent complete data collection were excluded. We also excluded cases with a preexisting score of more than 2 on the modified Rankin scale (mRS) and who lived dependently (17).

The study protocol was approved by the Ethics Committee of People's Hospital of Deyang City (Reference No. 2011-04-134). Written informed consent was obtained from all patients before they were enrolled or from their legal representative if the patient lost the capacity to give informed consent.

### Data Collection and Outcome

Baseline data on age, sex, admission delay, initial stroke severity assessed by the National Institutes of Health Stroke Scale (NIHSS) score, baseline systolic and diastolic blood pressure, serum glucose on admission, and vascular risk factors were collected and compared according to the stroke side (left or right hemisphere involvement). The potential etiology of LHI was classified as cardio-embolism or not according to the Trial of Org 10172 in Acute Stroke Treatment (TOAST) criteria (18). Two trained neurologists who were blinded to clinical information independently reviewed the imaging. A disagreement was resolved through discussion and consultation with a third neurologist. The presence of the HDMCAs was assessed on the pretreatment NCCT according to the following criteria (19): spontaneous visibility of the whole or part of horizontal segment of the MCA, the density of the MCA higher than that of the surrounding brain, disappearance on bone windows, unilaterality, and absence of subarachnoid bleeding. Early CT signs of hypodensity > 1/3 MCA territory was defined as the substantial involvement of ≥2 of the following four areas: frontal, temporal, parietal, or both basal ganglia and insula (20). The ASPECTS was assessed on the pretreatment NCCT (21). Final infarct territory on the following-up imaging was dichotomized into MCA territory and MCA plus (involvement of other vascular territories besides the MCA territory). MBE was defined as the development of clinical signs of herniation (including decease in consciousness and/or anisocoria), accompanied by a midline shift of ≥5 mm at the septum pellucidum or pineal gland with effacement of the basal cisterns on follow-up imaging, without other known/apparent causes of deterioration (22). Hemorrhagic transformation during hospitalization was classified as hemorrhagic infarct (HI) and parenchymal hematoma (PH) based on follow-up CT or MRI according to the recommendations of the European Cooperative Acute Stroke Study (ECASS) criteria (23).

In-hospital treatments analyzed in our study included intravenous thrombolysis (IVT), endovascular interventions (EVT), DHC, mechanical ventilation, and osmotic agents (such as mannitol). IVT or EVT was performed according to the Chinese guidelines, and the inclusion and exclusion criteria were similar to those of the American guideline (24, 25). The final treatment decision was made in consultation with the neurologist and the patient's family. Post-EVT recanalization was evaluated on the digital subtraction angiography according to the Thrombolysis in Cerebral Infarction (TICI) grading system. Successful recanalization was defined as a TICI grade of 2b or 3 (26, 27). DHC was considered for patients with LHI with significant neurological deterioration and MBE, and the decision was finally made in consultation with neurosurgeons and the patient's family. Stroke-related complications, including both neurological and medical complications during hospitalization, were reviewed by data collectors who were not aware of the study from hospital records when the patient was discharged (10). Neurological complications included MBE, hemorrhagic transformation, post-stroke seizures/epilepsy, central hyperthermia, and recurrent stroke, whereas medical complications included composite of cardiovascular events, pneumonia, urinary tract infection, gastrointestinal bleeding, electrolyte disorder, acute renal failure, urinary incontinence, hypoalbuminemia, deep venous thrombosis, and bedsore, which have been elaborated in our previous study (10). Composite of cardiovascular events in our study was defined as a composite of myocardial infarction, or acute heart failure, or any sudden cardiac death (28).

Patients were followed-up at 90 days after stroke onset by using questionnaires *via* a telephone interview or by mail. The primary outcome measures in our study were 1-month mortality, 3-month mortality, and unfavorable outcome [defined as an mRS score of 4 to 6 (17)].

### Statistical Analyses

The primary objective of our work was to test whether hemispheric side of LHI influenced the incidence of stroke-related complications and clinical outcomes. Baseline characteristics, in-hospital treatment, stroke-related complications, and outcomes were compared between patients with LHI with left-side and right-side lesions. Intergroup differences in categorical variables were assessed for significance using the χ^2^ tests or Fisher's exact tests, whereas differences in continuous variables were assessed using Student's *t*-tests or the Mann-Whitney *U*-test. Univariate analysis was performed to test variables that might affect the occurrence of stroke-related complications and outcomes. The included variables were: (1) age, (2) baseline NIHSS score, (3) vascular risk factors surveyed in our study, (4) imaging characteristics, (5) in-hospital treatment. The 3-month survival was estimated by the Kaplan–Meier method and a log-rank test was used for survival comparisons between patient groups. Multivariate analyses were performed to identify the association between the lateralization and the occurrence of stroke-related complications and outcomes, *via* adjusting for potential confounders (variables with *p* < 0.1 in univariate analyses). The 95% CI were calculated to describe the precision of the estimates. All statistical analysis was performed using SPSS v21.0 (SPSS, Chicago, IL). Two-sided *p* < 0.05 was considered to be statistically significant.

## Results

**Figure 1:**
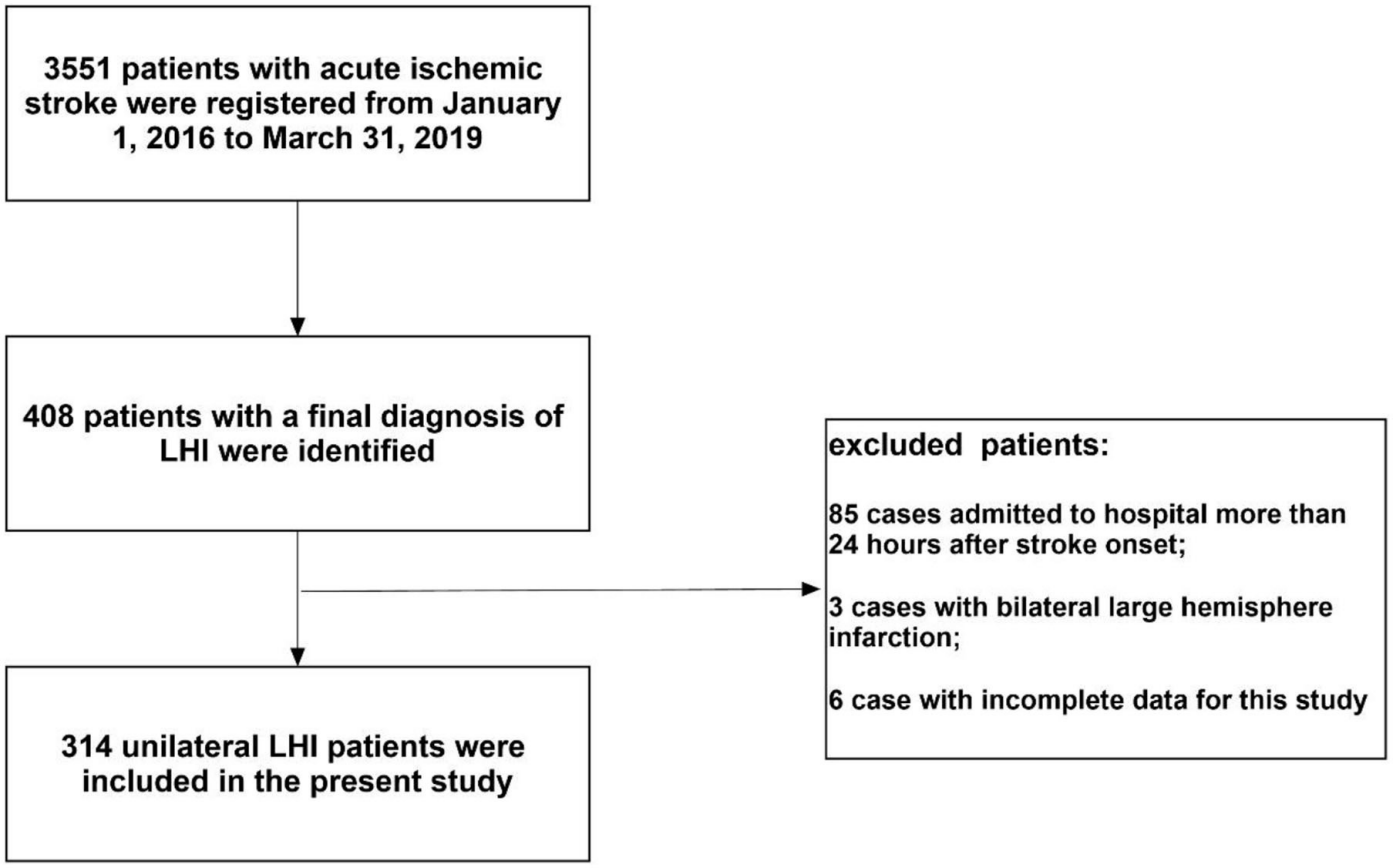
Flow diagram of included and excluded patients.

During the study period, 3,551 patients with AIS were registered. Of those patients, 314 (8.8%) unilateral patients with LHI admitted with 24 h were enrolled in the present study [mean age: 68.1 ± 14.8 years; 181 (57.6%) men; median NIHSS score on admission: 20]. A flow diagram of included and excluded patients is provided in Figure 1. All patients with LHI received CT scan at least one time and 156 (49.7%) patients received MRI. Among the enrolled patients, 171 (54.5%) were right hemisphere stroke and 143 (45.5%) were left hemisphere stroke. Sixty-one (19.4%) cases were treated with IVT and 46 (14.6%) were treated with EVT at the hyperacute stage. Thirty (9.6%) cases received DHC and 99 (31.5%) received mechanical ventilation during hospitalization. Five (1.6%) cases were lost to follow-up and 93 (29.6%) patients died at 30 days. Seven (2.2%) patients were lost to follow-up at 90 days (three in right-sided group and four in left-sided group). Among the entire cohort, 104 (33.1%) patients died and 221 (70.4%) patients had unfavorable outcome at 3 months.

### Hemispheric Differences in Characteristics and In-hospital Treatment

Compared with left hemisphere stroke, patients with LHI with right-side lesions had lower baseline NIHSS score (18 vs. 22, *p* < 0.001). Right hemisphere LHI showed higher rate of atrial fibrillation (69.0 vs. 52.4%, *p* = 0.003) and higher proportion of cardio-embolism (73.1 vs. 56.6%, *p* = 0.013) than the left. There was no difference in the mean age, gender, median admission delay, baseline blood pressure, baseline serum glucose, or other vascular risk factors between the two groups (all *p* > 0.05). Right hemispheric LHI had a lower median ASPECTS (six vs. seven, *p* = 0.004) and a higher rate of basal ganglia involvement on the pretreatment NCCT (70.2 vs. 58.0%, *p* = 0.025); however, the presence of HDMCAS, hypodensity > 1/3 of the MCA territory on baseline CT scan, and final infarct territory were comparable between the two groups (all *p* > 0.05). Then 21 (12.3%) cases with right hemisphere involvement and 25 (17.5%) cases with left hemisphere involvement were treated with EVT (*p* = 0.194) at the hyperacute stage. Among those patients, successful recanalization was achieved in 12 (57.1%) right-sided LHI patients and 20 (80.0%) left-sided patients. Patients with LHI with right-side involvement more frequently received DHC (14.0 vs. 4.2%, *p* = 0.003). There was no difference in the administration rate of IVT, mechanical ventilation, and osmotic therapy (all *p* > 0.05) (Table 1).

**Table 1:** Hemispheric differences in baseline characteristics and in-hospital treatment of LHI patients.

	**Right hemisphere**	**Left hemisphere**	***P-*value**
	**(*n* = 171)**	**(*n* = 143)**	
Age (years), mean ± SD	69.1 ± 14.2	66.9 ± 15.5	0.180^*^
Male, *n* (%)	91 (53.2)	90 (62.9)	0.083^‡^
Time from onset (h), median (range)	5 (3-11)	5 (3-14)	0.771^†^
Baseline NIHSS score, median (range)	18 (15-22)	22 (20-25)	**<0.001** ^†^
SBP on admission (mm Hg)	140.3 ± 24.5	143.1 ± 25.1	0.326^*^
DBP on admission (mm Hg)	83.0 ± 16.2	82.2 ± 13.9	0.659^*^
Baseline serum glucose (mmol/L)	8.2 ± 2.4	7.8 ± 2.8	0.259^*^
**Risk factors**, ***n*** **(%)**			
Hypertension	94 (55.0)	67 (46.9)	0.152^‡^
Diabetes mellitus	39 (22.8)	29 (20.3)	0.588^‡^
Dyslipidemia	36 (21.1)	30 (21.0)	0.987^‡^
Coronary heart disease	29 (17.0)	16 (11.2)	0.146^‡^
Atrial fibrillation	118 (69.0)	75 (52.4)	**0.003** ^‡^
Rheumatic heart disease	25 (14.6)	19 (13.3)	0.735^‡^
Current smoking	54 (31.6)	52 (36.4)	0.372^‡^
Alcohol consumption	43 (25.1)	33 (23.1)	0.670^‡^
Previous ischemic stroke/TIA	27 (15.8)	21 (14.7)	0.787^‡^
Previous ICH	2 (1.2)	2 (1.4)	0.857^‡^
TOAST classification, *n* (%)			**0.013** ^‡^
Large-artery atherosclerosis	20 (11.7)	25 (17.5)	
Cardio-embolism	125 (73.1)	81 (56.6)	
Other determined etiology	5 (2.9)	12 (8.4)	
Undetermined etiology	21 (12.3)	25 (17.5)	
HDMCAS, *n* (%)	51 (29.8)	34 (23.8)	0.230^‡^
Hypodensity > 1/3 MCA territory	51 (29.8)	33 (23.1)	0.191^‡^
ASPECTS, median (range)	6 (4-8)	7 (5-9)	**0.004** ^†^
Basal ganglia Involvement	120 (70.2)	83 (58.0)	**0.025** ^‡^
Final infarct territory			0.066^‡^
MCA	142 (83.0)	129 (90.2)	
MCA plus	29 (17.0)	14 (9.8)	
**Treatments**, ***n*** **(%)**			
Intravenous thrombolysis^**∧**^	34 (19.9)	27 (18.9)	0.823
Endovascular interventions^**∧**^	21 (12.3)	25 (17.5)	0.194
DHC^**∧**^	24 (14.0)	6 (4.2)	**0.003**
Mechanical ventilation^**∧**^	61 (35.7)	38 (26.6)	0.084
Osmotic agents^**∧**^	166 (97.1)	134 (93.7)	0.150

*SD, Standardized difference; NIHSS, National Institutes of Health Stroke Scale; SBP, systolic blood pressure; DBP, diastolic blood pressure; TOAST, Trial of Org 10172 in Acute Stroke Treatment; TIA, transient ischemic stroke; ICH, intracerebral hemorrhage; HDMCAS, hyperdensity in the middle cerebral artery (MCA); DHC, decompressive hemicraniectomy*.

* *Student t-test*.

† *Mann–Whitney U-test*.

‡ *χ2 test. The bold values indicates p < 0.05*.

∧ *Acute phase treatment*.

### Hemispheric Differences in the Incidence of Stroke-Related Complications

**Figure 2:**
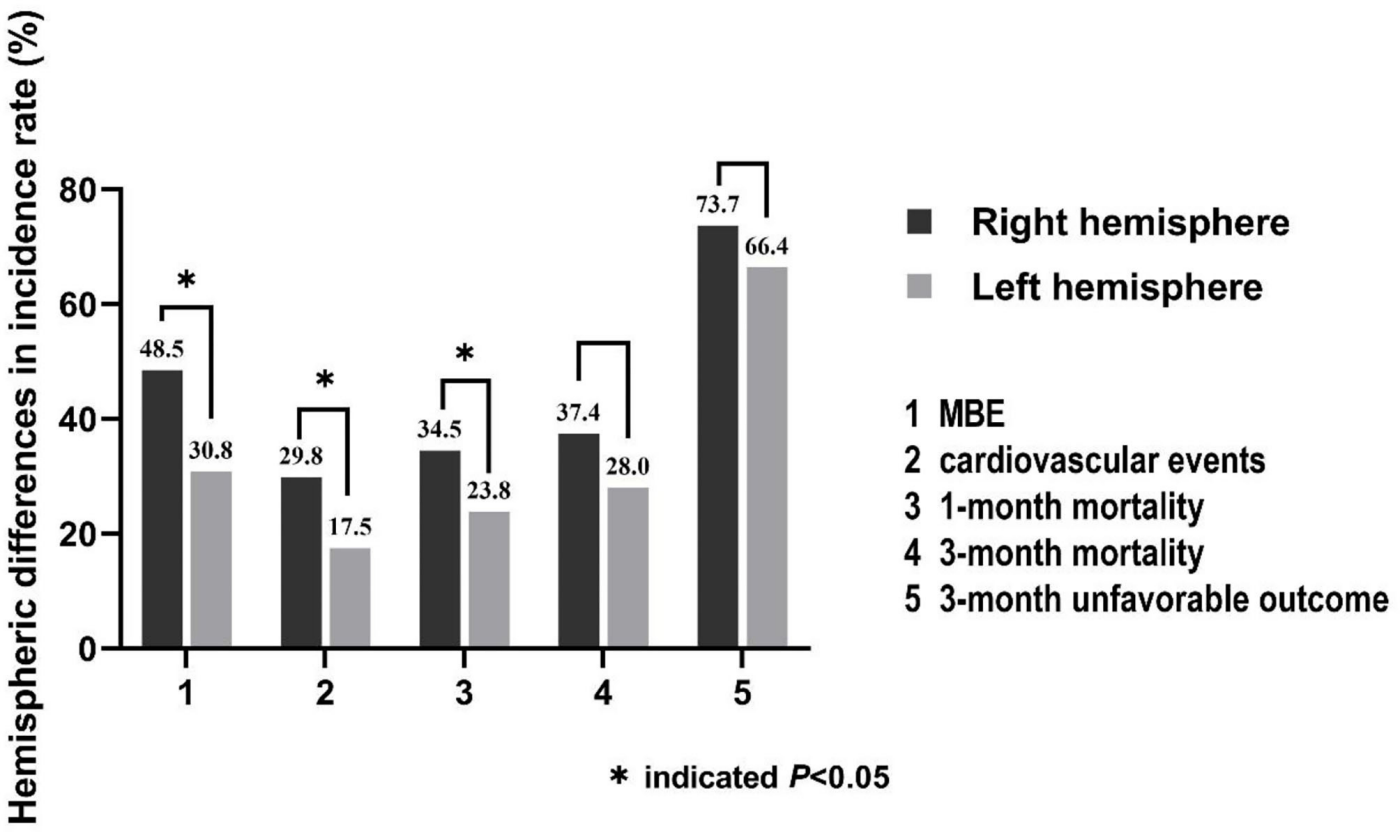
Hemispheric differences in the incidence rates of stroke-related complications (MBE and composite of cardiovascular events) and clinical outcomes.

Large hemisphere infarctions patients with right hemisphere involvement had higher incidence rates of MBE (48.5 vs. 30.8%, *p* = 0.001) and composite of cardiovascular events (29.8 vs. 17.5%, *p* = 0.011) during hospitalization than those with left-side lesions (Figure 2). However, no significant difference was found in the incidence rate of total hemorrhagic transformation (48.0 vs. 49.0%, *p* = 0.860), HI (18.1 vs. 18.9%, *p* = 0.864), and PH (29.8 vs. 30.1%, *p* = 0.962) between right and left hemisphere stroke patients. Meanwhile, there was no significant difference in the events rates of seizures/epilepsy, central hyperthermia, recurrent stroke, pneumonia, urinary tract infection, gastrointestinal bleeding, electrolyte disorder, acute renal failure, urinary incontinence, hypoalbuminemia, deep venous thrombosis, and bedsore between right and left hemisphere stroke patients (all *p* > 0.05) (Table 2). After adjusting for age, baseline NIHSS score and other confounders in multivariate analyses, right hemisphere involvement was independently associated with increased risk of MBE (adjusted OR 2.37, 95% CI 1.26–4.43, *p* = 0.007) and composite of cardiovascular events (adjusted OR 2.04, 95% CI 1.12–3.72, *p* = 0.020) (Table 3).

**Table 2:** Hemispheric differences of and stroke-related complications during hospitalization in LHI patients.

	**Right hemisphere**	**Left hemisphere**	***P-*value**
	**(*n* = 171)**	**(*n* = 143)**	
**Neurological complications**, ***n*** **(%)**			
Malignant brain edema	83 (48.5)	44 (30.8)	**0.001**
Hemorrhagic transformation	82 (48.0)	70 (49.0)	0.860
HI	31 (18.1)	27 (18.9)	0.864
PH	51 (29.8)	43 (30.1)	0.962
Seizures/epilepsy	11 (6.4)	13 (9.1)	0.377
Central hyperthermia	23 (13.5)	10 (7.0)	0.063
Recurrent stroke	4 (2.3)	1 (0.7)	0.381^*^
**Medical complications**, ***n*** **(%)**			
Composite of cardiovascular events	51(29.8)	25(17.5)	**0.011**
Pulmonary infection	129 (75.4)	109 (76.2)	0.871
Urinary tract infection	36 (21.1)	30 (21.0)	0.987
Gastrointestinal bleeding	71 (41.5)	62 (43.4)	0.743
Electrolyte disturbance	105 (61.4)	80 (55.9)	0.327
Acute renal failure	43 (25.1)	27 (18.9)	0.184
Hypoalbuminemia	84 (49.1)	70 (49.0)	0.976
Urinary incontinence	35 (20.5)	28 (19.6)	0.845
Deep venous thrombosis	16 (9.4)	15 (10.5)	0.738
Bedsore	4 (2.3)	3 (2.1)	1.000^*^
**Clinical outcomes**, ***n*** **(%)**			
1-month mortality	59 (34.5)	34 (23.8)	**0.036**
3-month mortality	64 (37.4)	40 (28.0)	0.086
3-month unfavorable outcome	126 (73.7)	95 (66.4)	0.196

* *Fisher's exact test. The bold values indicates p < 0.05*.

**Table 3:** Univariate and multivariate analyses for the association between right hemisphere involvement and stroke-related complications and outcome in LHI patients.

	**Univariate analysis**		Multivariate analysis^*^	
	**OR (95%CI)**	***P-*value**	**OR (95%CI)**	***P-*value**
Malignant brain edema	2.12 (1.33-3.38)	**0.002**	2.37 (1.26-4.43)^a^	**0.007**
Composite of cardiovascular events	2.01 (1.17-3.45)	**0.012**	2.04 (1.12-3.72)^b^	**0.020**
1-month mortality	1.70 (1.03-2.81)	**0.037**	1.42 (0.73-2.73)^c^	0.302
3-month mortality	1.52 (0.94-2.47)	0.087	1.21 (0.62-2.34)^c^	0.574
3-month unfavorable outcome	1.39 (0.84-2.29)	0.197	1.20 (0.59-2.45)^d^	0.616

* *Adjusted for variables which had a significant association with corresponding stroke-related complications and clinical outcomes in univariate analysis*.

*OR, odds ratios; CI,confidence intervals*.

a *Adjusted for age, baseline NIHSS score, baseline diastolic blood pressure, TOAST classification, hypodensity > 1/3 MCA territory on baseline CT scan, baseline ASPECTS, basal ganglia involvement, involvement of other vascular territories besides the MCA territory, and EVT, which had a potential association with malignant brain edema in univariate analysis*.

b *Adjusted for age, sex, baseline NIHSS score, predisposing vascular risk factors (hypertension, diabetes mellitus, dyslipidemia, previous ischemic stroke/TIA, current smoking and alcohol consumption), prior-to-stroke heart diseases (coronary heart disease, atrial fibrillation, rheumatic heart disease), and acute reperfusion therapies (IVT and EVT), which had a potential association with composite of cardiovascular events in univariate analysis*.

c *Adjusted for age, baseline NIHSS score, baseline systolic blood pressure, baseline serum glucose, hypodensity > 1/3 MCA territory on baseline CT scan, ASPECTS, involvement of other vascular territories besides the MCA territory, EVT, DHC and mechanical ventilation, which had a potential association with death of LHI patients in univariate analysis*.

d *Adjusted for age, baseline NIHSS score, baseline systolic blood pressure, baseline serum glucose, hypodensity > 1/3 MCA territory on baseline CT scan, ASPECTS, involvement of other vascular territories besides the MCA territory, EVT, DHC and mechanical ventilation, which had a potential association with death of LHI patients in univariate analysis. The bold values indicates p < 0.05*.

Since the occurrence of MBE is closely related to whether EVT was carried out, the recanalization status (successful recanalization or not), and also whether there was hemorrhagic transformation, we conducted a sensitivity analysis including EVT, successful recanalization, and hemorrhagic transformation in the multivariate analysis. After adjusting for confounders, right-sided stroke was still an independent factor associated with increased risk of MBE in LHI patients (adjusted OR 3.12, 95% CI 1.55–6.29, *p* = 0.001).

### Hemispheric Differences in the Outcomes

**Figure 3:**
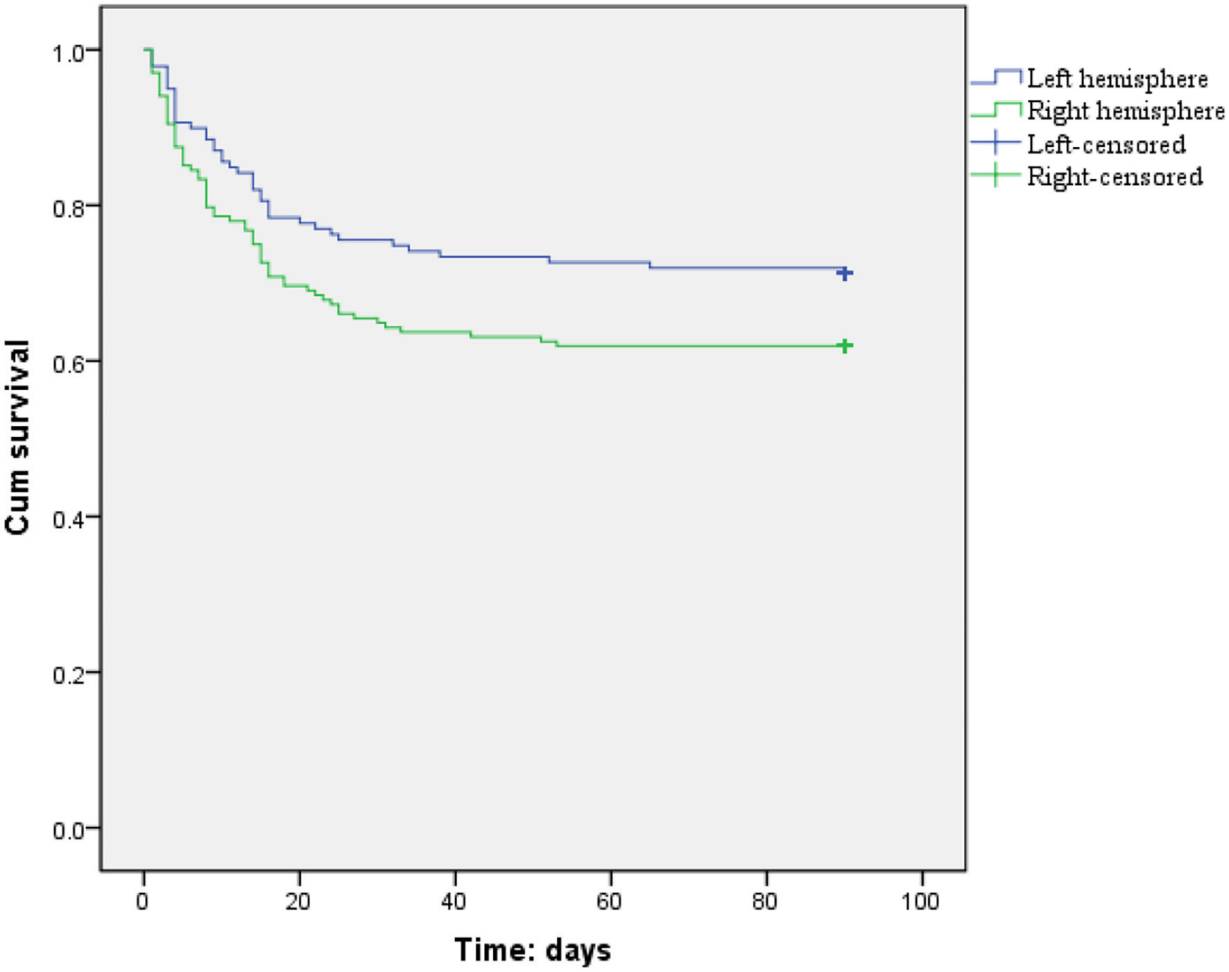
Three-month survival curves for LHI patients estimated by Kaplan–Meier analysis (patients with right or left hemisphere involvement, *p* = 0.071, log rank test).

The incidence rate of 1-month mortality (34.5 vs. 23.8%, *p* = 0.036) was higher among LHI patients with right hemisphere involvement, but there were no hemispheric differences in the incidence rates of 3-month mortality (37.4 vs. 28.0%, *p* = 0.086) and 3-month unfavorable outcome (73.7 vs. 66.4%, *p* = 0.196) (Figure 2; Table 2). Meanwhile, the 3-month survival rate of the right hemispheric LHI estimated by Kaplan-Meier Method was not significantly higher than the left-sided (*p* = 0.071, log-rank test; Figure 3). After adjusting for age, baseline NIHSS score, baseline systolic blood pressure, baseline serum glucose, hypodensity > 1/3 MCA territory on baseline CT scan, ASPECTS, involvement of other vascular territories besides the MCA territory, EVT, DHC, and mechanical ventilation, which had a potential association with death and unfavorable outcomes of LHI patients in univariate analysis, right hemisphere involvement was not independently associated with 1-month mortality, 3-month mortality, and 3-month unfavorable outcome of LHI patients (all *p* > 0.05, see in Table 3).

## Discussion

Several studies have reported the left-right propensity of cardioembolic infarcts, but no consensus has been reached so far (29–34). Although the left hemisphere propensity of cardiogenic emboli has been reported (29, 30), some researches supported the right-sided propensity of cardio-embolism, especially those associated with atrial fibrillation (31, 32). A recently published work indicated that bovine aortic arch was associated with left hemisphere laterality of cardioembolic stroke compared with standard arches (33). Another work suggested that there was a trend toward right-sided lesions in patients with standard arches, but no significant difference in cardio-embolic stroke laterality of patients with bovine arches was demonstrated (34). In this work, we found that LHI patients with right hemisphere involvement had higher frequency of atrial fibrillation and cardio-embolism, supporting the right-sided propensity of cardio-embolism in LHI patients. It is worth noting that although there were 1.6% (5/314) left-handed patients (all with right hemisphere infarction) in the present cohort, there was no significant difference in the presence of left-handedness among LHI patients with a stroke etiology of cardiogenic embolism or not, supporting that the right-sided propensity of cardio-embolism in LHI patients was independent of whether it was a non-dominant hemisphere or not. Our results could be explained by the fact that the brachiocephalic artery is the first branch off the aortic arch, has the largest ostium, and heads upward and parallel to the direction of the ascending aorta. So large-sized cardiogenic emboli would therefore have a higher propensity to enter the brachiocephalic artery (which supplies the right common carotid and right subclavian) rather than the left common carotid artery, which arises second from the aortic arch and has an orientation perpendicular to it (33–35). Further study is warranted to determine whether there is a difference in the laterality of cardiogenic infarct depending on the size of embolic particles besides the aortic arch branching pattern.

Previous works have suggested that higher baseline NIHSS score was one of the early predictors for MBE after ischemic stroke (36). Meanwhile, the NIHSS score are also weighted toward left hemisphere lesions (11). As a result, right hemisphere infarctions can be deemed to be less severe than left-sided, such that physicians and surgeons might be less aggressive in treatment. However, our study found that patients with LHI with right hemisphere involvement had higher incidence rate of MBE (48.5 vs. 30.8%) than those with the left. Multivariate analysis suggested that right hemisphere stroke (OR 2.37, 95 % CI 1.26-4.43) was an independent risk factor of MBE in patients with LHI. Although there was a lower successful recanalization rate of right-sided stroke patients following EVT in our cohort (57.1 vs. 80.0%), multivariate analysis including EVT, successful recanalization, and hemorrhagic transformation also identified right hemisphere involvement as an independent risk factor of MBE in LHI patients. These results are consistent with the result of a systematic review, which included a total of 73 relevant studies and concluded that “malignant” MCA infarction appears to be more common in the right hemisphere (12). These results can be explained because the right hemisphere plays a crucial role in cardiovascular regulation due to autonomic nervous system lateralization, leading to greater alterations of norepinephrine levels and sympathetic activation after right hemispheric insular infarction than the left (37). Increased levels of norepinephrine can result in vasoconstriction, increasing the permeability of blood–brain barrier and the levels of extracellular glutamate by stimulation of β-adrenoreceptor, creating an intracellular Ca^2+^ and Na^+^ osmotic gradient and thus attracting extracellular fluid into the cell resulting in the development of cytotoxic edema (12, 37, 38). Moreover, increased levels of norepinephrine can stimulate the α-1 adrenergic receptors in the supraoptic nucleus of the hypothalamus and thereby enhance the release of vasopressin, leading to the downregulation of aquaporin-4 and increasing the plasma membrane permeability (12, 39). Vasopressin can also participate in the ischemic cascade, leading to neuroinflammation, necrosis, and apoptosis that might be another factor in the asymmetrical development of MBE (12, 39).

Several clinical works indicate that the effect on the cardiovascular system depends on the stroke lateralization (40–43). It has been reported that when brain damage affects the right insular cortex, the consequences on cardiac function are more deleterious and more frequent (40–42). Conversely, another work indicates that left insular stroke appears to be an independent predictor of severe cardiovascular consequences such as cardiac death, acute myocardial infarction, angina, or heart failure (41). The phenomenon of stroke lateralization has also been observed in experimental models (44, 45). It has been demonstrated that right MCA occlusion (MCAO) in rats had more cardiovascular consequences than the left MCAO (44, 45). In our work, we found that right-sided LHI patients had higher rate of composite of cardiovascular events (29.8 vs. 17.5%) during hospitalization than the left-sided. After adjusting for age, sex, baseline NIHSS score, predisposing vascular risk factors (hypertension, diabetes mellitus, dyslipidemia, previous ischemic stroke/TIA, current smoking, and alcohol consumption), prior-to-stroke heart diseases (coronary heart disease, atrial fibrillation, rheumatic heart disease), and acute reperfusion therapies (IVT and EVT), which had a potential association with composite of cardiovascular events, right hemisphere involvement was still an independent risk factor of composite of cardiovascular events in patients with LHI (OR 2.04, 95% CI 1.12–3.72). This result could be explained by the fact that right hemisphere stroke, especially those with insular cortex involvement, is responsible for autonomic disturbances and triggers inflammatory processes including the release of cytokines such as monocyte chemoattractant protein-1, C-reactive protein, growth differentiation factor, leading to an increased risk of cardiac death, acute myocardial infarction, and heart failure (46).

Our work found that although the right-sided LHI had higher incidence rate of 1-month mortality (34.5 vs. 23.8%) in univariate analysis, multivariate analysis suggested that stroke lateralization was not an independent predictor of mortality and unfavorable outcome in LHI patients. Previous studies have not reached a consensus on the impact of the stroke hemisphere on outcomes of AIS (12–15). A cohort study indicated that left-hemispheric AIS were often associated with a worse outcome than their right-hemispheric counterparts (14). However, several studies suggested an association between right hemisphere involvement and higher risk of death and unfavorable functional outcome in AIS patients, especially for those with mild/moderate strokes (13, 47). The results of our work are in line with previous published systematic review and metaanalysis, which indicated that stroke lateralization was not an independent predictor of mortality and unfavorable outcome and did not modify the treatment effect of MCA territorial infarction (12, 15).

### Limitations

The results of the present work should be interpreted with caution given its limitations. First, we conducted a retrospective study using the prospective data from the Deyang stroke registry, and so we could not provide information related to inflammatory cytokines and markers of myocardial injury such as C-reactive protein, troponins, or other acute-phase protein, and further studies are needed to explore this issue. Second, it was a single hospital-based study, with limited generalizability. Some patients with severe LHI might not be hospitalized, especially those who died before being admitted to the hospital, and so we could not exclude inclusion bias in this study. Third, follow-up in our study was performed by telephone interview or a mailed questionnaire instead of a clinic visit which may result in reporting bias.

## Conclusions

We identified that patients with LHI with right hemisphere involvement had higher frequency of atrial fibrillation and cardio-embolism. Right hemisphere involvement was independently associated with increased risk of MBE and composite of cardiovascular events during hospitalization, whereas hemispheric difference was not an independent predictor of mortality and unfavorable outcome in LHI patients. Implement of early pharmacological or non-pharmacological intervention for autonomic restoration might improve the outcome of right-sided LHI patients, especially those with insular cortex involvement.

## Data Availability Statement

The raw data supporting the conclusions of this article will be made available by the corresponding author on reasonable request.

## Ethics Statement

The studies involving human participants were reviewed and approved by the Ethics Committee of People's Hospital of Deyang City. The patients/participants provided their written informed consent to participate in this study.

## Author Contributions

JL and PZ collected, analyzed, and interpreted the data, as well as drafted the manuscript. YL and WC participated in data interpretation and revised the manuscript. XY participated in study conception and design. CW contributed substantially to study design and supervision, data interpretation and manuscript writing. All authors critically revised the manuscript for important intellectual content and approved the final manuscript.

## Funding

This research was funded by Universal Application Program, Health and Family Planning Commission of Sichuan Province (17PJ084), the Science and Technology Research Foundation of Deyang City (2020SZZ069), and Applied Basic Research Program, Science and Technology Department of Sichuan Province (No. 2018JY0389) in China.

## Conflict of Interest

The authors declare that the research was conducted in the absence of any commercial or financial relationships that could be construed as a potential conflict of interest.

## Publisher's Note

All claims expressed in this article are solely those of the authors and do not necessarily represent those of their affiliated organizations, or those of the publisher, the editors and the reviewers. Any product that may be evaluated in this article, or claim that may be made by its manufacturer, is not guaranteed or endorsed by the publisher.
